# Functional components profile and glycemic index of kidney beans

**DOI:** 10.3389/fnut.2022.1044427

**Published:** 2022-11-02

**Authors:** Shengshu Xu, Likang Qin, Muhammad Mazhar, Yong Zhu

**Affiliations:** ^1^School of Liquor and Food Engineering, Guizhou University, Guiyang, China; ^2^College of Life Sciences, Guizhou University, Guiyang, China

**Keywords:** kidney beans, α-amylase inhibitor, resistant starch, glycemic index, type II diabetes

## Abstract

Low glycemic index (GI) diet has been considered as a strategy for type II diabetes patients. In the present study, the phenolics profile, α-amylase inhibitor activities, starch composition as well as the glycemic index of seven varieties of kidney beans were studied. An enzymatic inhibitory reaction model was employed to determine the α-amylase inhibitor activity, and the *in vitro* digestion model coupled with the 3, 5-dinitrosalicylic acid colorimetry method was adopted to evaluate the starch composition and glycemic index. The results showed that gallic acid was dominant in kidney beans, and the colored beans contained more phenolics than the white ones. In addition, the α-amylase inhibitor activities of kidney beans ranged from 1.659 ± 0.050 to 4.162 ± 0.049 U/g DW, among which the Y2 variety was the top-ranked. Furthermore, kidney beans starch demonstrated brilliant resistance to digestion with the contribution of resistant starch to total starch between 70.90 ± 0.39% and 83.12 ± 0.42%. Eventually, these kidney beans were categorized as low GI foods, which ranged from 32.47 ± 0.13 to 52.99 ± 0.56, the resistant starch makes dominant contribution to the low GI. These results indicate that kidney beans can be served as ingredients in functional low GI foods.

## Introduction

Type II diabetes is a principal chronic disease that threatens human health which is initiated by the sugar metabolism disorder (1, 2). Accumulating evidences have shown that diets with a low glycemic index (GI) are efficient strategies to reduce the complications associated with type II diabetes (1, 3). The GI indicates the blood glucose response after intake a food with a specific amount of accessible carbohydrates compared with a reference food (4, 5).

Kidney bean (*Phaseolus vulgaris* L.) is one of the edible beans which includes red kidney bean, white kidney bean, red speckled kidney bean, and black kidney bean. They contain abundant phenolics, functional proteins, as well as other active ingredients that present antioxidant, hypoglycemic, and hypolipidemic properties (6, 7). Gallic acid, ferulic acid, catechin, *p*-hydroxybenzoic acid have been identified in beans (6, 8–10). Apart from antioxidant activity, phenolic compounds may also play a role in inhibiting α-glucosidase and lipase activities (10, 11). Kidney bean contains a glycoprotein named as α-amylase inhibitor (α-AI), which can inhibit starch digestion (12, 13). Previous studies showed that the α-AI proteins extracted from beans showed a strong inhibitory activity on α-amylase (14), and the ingestion of white bean extracts that contain α-AI can mitigate the obesity and regulate the gut microbiota of obese rats (15), furthermore, the resistant starch (RS) and phenolics were the dominant components that contribute to the low GI of starchy foods (16). As such, these active ingredients may possess the potential to control postprandial blood glucose levels (12, 17), which provide health benefits to the patients with type II diabetes (16, 18, 19).

In the present study, the functional properties of kidney beans were evaluated in aspects of phenolics and starch composition, α-AI activity as well as the potential blood glucose response, which provided the fundament for the application of kidney beans in low GI foods.

## Materials and methods

### Sample preparation

**FIGURE 1:**
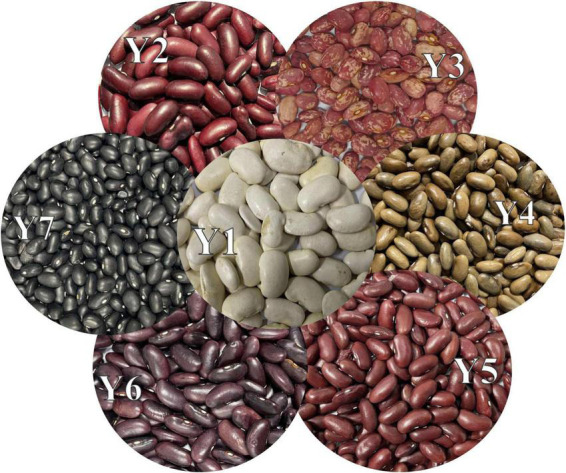
Seven varieties of kidney beans.

Seven varieties kidney beans with different coat colors—Weining white kidney bean, Biyun no.7, Qian yundou no.1, YJ009727A (serial number), Biyun1902, Biyun no.6, and Biyun no.3—were provided by the Guizhou Bijie Institute of Agricultural Sciences of China, which were labeled Y1, Y2, Y3, Y4, Y5, Y6, and Y7, respectively (Figure 1). These kidney beans were crushed into powder using a high-speed universal crusher, and they were then screened using a 40-mesh sieve and subsequently stored at −20^°^C for further analysis. White bread was purchased from a local supermarket.

### Chemicals

Pepsin (source: gastric mucosa of a pig) and gallic acid were purchased from Sigma-Aldrich Ltd (St. Louis, Mo, USA). 3, 5-Dinitrosalicylic acid (DNS) was purchased from Sinopharm Chemical Reagent Co., Ltd (Shanghai, China). Acetone and sodium carbonate were purchased from Chuandong Co., Ltd (Chongqing, China). HPLC-grade acetonitrile and acetic acid were purchased from Aladdin company (Shanghai, China). Glucose was purchased from Yong Zhu Da Chemical Reagent Co., Ltd (Tianjin China). Rutin, chlorogenic acid, 2, 3, 4-trihydroxybenzoic acid, *p*-hydroxybenzoic acid, catechin and ferulic acid were purchased from Macklin Bioch. Co., Ltd (Shanghai, China). Folin-Ciocalteu’s phenol reagent and the α-amylase enzyme (type: pancreatic, source: bacillus) were purchased from Solarbio Science & Tech. Co., Ltd (Beijing China). Other reagents were purchased from Chengdu Jinshan Chemical Reagent Co., Ltd (Chengdu, China).

### Extraction of phenolics

Four grams kidney bean powder was extracted using 15 mL 80% chilled acetone for 15 min, the centrifugation (8,000 g, 5 min) (CenLee16R, Hunan Cenlee Scientific Instrument Co., Ltd., Hunan, China) was performed prior to collecting supernatant, and residues were extracted twice according to the above procedure. The supernatant was evaporated to 5 mL using a rotary evaporator at 45^°^C, and the volume was then constituted to 10 mL with deionized water. Lastly, the extract was stored in a refrigerator at −20^°^C for subsequent experiments (20). Each kidney bean powder was extracted in triplicate.

### Determination of total phenolics

The total phenolics was determined by referring to previous studies (20, 21). A 100 μL amount of the diluted extract (or gallic acid standard) was pipetted into a 10 mL test tube, then, 400 μL of deionized water and 100 μL of Folin’s phenol reagent were added into the tube prior to a 6-min static reaction (at room temperature). The mixture was then mixed with 1 mL of seven percent sodium carbonate solution and 0.8 mL of deionized water, and the reaction continued for 90 min under dark conditions. Next, 200 μL of the reaction solution was pipetted into 96-well microplates and measured at 760 nm. The standard curve was produced by plotting the standard concentration and absorbance. The total phenolics of the kidney bean extract was calculated from the standard curve and expressed as the milligram gallic acid equivalents per 100 grams dry weight of the kidney bean (mg GAE/100 g DW).

### Determination of total flavonoids

The total flavonoids was determined using the aluminum salt method (20). A 0.5 mL amount of extract (or rutin standard) was pipetted into a 10 mL test tube followed by 2.25 mL of deionized water and 0.15 mL of 5% sodium nitrite solution addition. After a 6 min reaction, 0.3 mL of 10% AlCl_3_.6H_2_O solution was added, and the solution was reacted for 5 min. Then, 1 mL of 1 mol/L NaOH solution was mixed prior to reacting for 15 min. Finally, 200 μL of reaction solution was pipetted into the 96-well microplates, and a microplate reader (SPECTRA MAX 190, Molecular Devices, Silicon Valley, America) was used to measure the absorbance at 510 nm. The standard curve was plotted from the standard concentration and the corresponding absorbance, and the total flavonoids in the kidney bean extract was calculated from the standard curve and expressed as milligram rutin equivalents per 100 grams dry weight of the kidney bean (mg RE/100 g DW).

### Determination of phenolic compounds composition

The phenolic compounds composition was determined using HPLC (20, 22). An Agilent Technologies 1260 Infinity system coupled with an Agilent ZORBBAX-C18 column (250 mm × 4.6 mm, 5 μm) were employed. A 1 mL amount of extract was pipetted into brown autosampler vials after being filtered through a 0.45 μm membrane. Acetonitrile and a 1% glacial acetic acid solution were employed as mobile phases A and B, respectively. The following gradient elution program was adopted: 0–5 min, 5–15% A; 5–35 min, 15–35% A; 35–40 min, 35–45% A; 40–50 min, and 45–5% A. The injection volume was 20 μL, the flow rate was 1.0 mL per minute, and the column temperature was 30^°^C. An ultraviolet absorption detector was used to detect individual phenolic compounds at 280 nm. The standard curves of the individual phenolic compounds were plotted from the concentrations and corresponding peak areas, and the peaks were identified using the retention time and quantified from the corresponding standard curve. The contents of individual phenolic compounds were expressed as milligram per 100 grams dry weight of the kidney beans (mg/100 g DW).

### Determination of α-amylase inhibitor activity

A mixture of kidney bean powder (4 g) and 20 mL of deionized water was reacted in an oscillating water bath (TS-100C, TENSUC, Shanghai, China) at 25^°^C for 2 h. Then, the supernatant was collected via centrifugation (8,000 g, 4^°^C, 30 min) (CenLee16R, Hunan Cenlee Scientific Instrument Co., Ltd., Hunan, China), and the volume was subsequently constituted to 20 mL using deionized water. The water extract was immediately subjected to α-AI activity analysis. Each of the kidney beans powder was extracted in triplicate (4).

The α-AI activity was determined according to the method described by previous studies (4, 13) with minor modifications. The water extract was properly diluted to keep the inhibition rate (IR) of α-amylase below 50%. A mixture comprising 0.25 mL of the diluted water extract, 0.25 mL of α-amylase solution (1 U/mL) and 0.5 mL of PBS (pH 6.9) was reacted in an oscillating water bath at 37°C for 10 min. Then, 0.5 mL 1% (W/W) soluble starch solution was added, and a 5 min reaction was allowed in an oscillating water bath at 37°C. Prior to being heated in a boiling water bath, 1 mL DNS solution was added into the mixture, then it was cooled in a cold water bath, the mixture volume was eventually constituted to 20 mL using deionized water, the absorbance of the mixture was measured at 540 nm using an ultraviolet spectrophotometer (L5S, Shanghai yidian analytical instrument co. LTD, Shanghai, China). The following groups that were subjected to no water extract, neither water extract nor α-amylase solution and no α-amylase solution–were regarded as the blank, blank control, and inhibitory control groups, respectively. The IR of α-AI to α-amylase was calculated using the following formula:


$$\text{IR}=\left(1-\frac{A_{3}-A_{4}}{A_{1}-A_{2}}\right)\times 100\%$$

where *A1* represents the absorbance of the blank group, *A2* represents the absorbance of the blank control group, *A3* represents the absorbance of the experimental group, and *A4* represents the absorbance of the inhibitory control group.

The amount of α-amylase that catalyzed the formation of 1 mol glucose per minute at 37°C with a pH of 6.9 was quantified as an activity unit (U). The quantity of α-AI that inhibited the catalyzing of 1 mol glucose formation per minute in the α-amylase catalytic starch hydrolysis reaction at 37°C with a pH of 6.9 was quantified as an activity unit (U). α-AI-specific activity (U/g) was defined as the α-AI activity unit in one gram of kidney bean and was calculated according to the following formula:


α-AI=(I⁢R×1×n×V)/m

where *1* is the activity of the α-amylase solution (U/mL), *n* represents the dilution times of water extract, *V* represents the total volume of the water extract (mL), and *m* represents the mass of the kidney bean powder (g).

### Determination of total starch

The total starch (TS) in the kidney beans was determined according to the National Food Safety Standards of China, GB 5009.9 (23). The kidney bean powder underwent a dual extraction procedure to remove fat and soluble sugar in triplicate, and the residues were then subjected to continuous processes that include gelatinization, saccharification, acid hydrolysis, and neutralization prior to glucose content determination via the alkaline copper tartrate solution titration method.

### *In vitro* simulated digestion

An aliquot of kidney bean powder containing 50 mg of starch was mixed with 2 mL of deionized water, and the mixture was subsequently heated in a boiling water bath (10 min) to gelatinize the starch. Then, it was cooled to room temperature prior to being mixed with 10 mL of HCl-KCl buffer (0.1 mol/L, pH = 1.5) and 0.2 of mL pepsin solution (1 mg/mL) in an oscillating water bath (40^°^C, 1 h) (TS-100C, TENSUC, Shanghai, China). The digestive mixture was then treated with 15 mL of PBS buffer (pH = 6.9) and 5 mL of α-amylase (2.6 U) to carry out the 180 min enzymatic digestion in an oscillating water bath at 37°C. The aliquots (1 mL) of mixture that have been digested within 0, 20, and 120 min were used to determine the starch properties (24, 25), and the other aliquots of mixture that have been digested within 0, 30, 60, 90, 120, 150, and 180 min were used for glycemic index determination. Reference substance (white bread) was subjected to the above procedure except the gelatinization process, and tests were performed in triplicate (4, 26). The aliquots of the digested mixture were boiled to inactivate enzyme activities and subsequently cooled using a cold water bath. The supernatant was then collected via centrifugation (8,000 g, 4^°^C, 10 min) (CenLee16R, Hunan Cenlee Scientific Instrument Co., Ltd., Hunan, China) and eventually subjected to glucose content determination. The above procedures were performed on the kidney bean powder in triplicate.

### Determination of glucose content

The DNS colorimetry was adopted to determine the glucose content (27). The mixture consisted of 0.2 mL of supernatant and 0.2 mL of DNS solution was boiled for 6 min in a water bath and subsequently cooled to room temperature with running water. The volume of the reaction mixture was constituted to 2 mL using deionized water, and the absorbance of the mixture was eventually measured using a spectrophotometer (L5S, Shanghai yidian analytical instrument co. LTD, Shanghai, China) at 540 nm.

### Calculation of starch composition

The contents of rapidly digestible starch (RDS), slowly digestible starch (SDS) and resistant starch (RS) were calculated by referring to the previous studies (24, 25). The following equations were used:


$$RDS\left(\%\right)=\left[\left(C_{20}-C_{0}\right)\times V\times 0.9/50\right]\times 100$$

$$SDS\left(\%\right)=\left[\left(C_{120}-C_{20}\right)\times V\times 0.9/50\right]\times 100$$

RS(%)=1-[(RDS+SDS)/50]×100

where *C*_0_ is the free glucose concentration before the enzymatic hydrolysis (mg/mL), *C*_20_ is the concentration of glucose released within 20 min (mg/mL), *C*_120_ is the concentration of glucose released within 120 min (mg/mL), *V* is the initial volume (mL) of the enzymatic hydrolysis reaction system, *0.9* is the conversion factor of glucose to starch, and *50* is the starch content (mg) in the measured sample.

### Calculation of glycemic index

The glycemic index of kidney bean was calculated by referring the previous studies (4, 26, 28). The digestion time (min) and starch hydrolysis rate (SR) were employed as the abscissa and the ordinate, respectively, to plot the starch hydrolysis curve. The SR was calculated using the following equation:


$$S\,R=\left[\left(C_{t}-C_{0}\right)\times V\times 0.9/50\right]\times 100\%$$

where *C*_*t*_ is the glucose content at time *t* (min) of the enzymatic hydrolysis, *C*_0_ is the glucose content at the beginning (0 min) of the enzymatic hydrolysis, *V* is the initial volume (mL) of the enzymatic hydrolysis reaction system, *0.9* is the conversion factor of glucose to starch, and *50* is the starch content (mg) in the measured sample. The kinetic constant (*K*) and the maximum glucose content (C_∞_) (mg/mL) were calculated from the following equation:


$$\left(C_{t}-C_{0}\right)=C\times \left[1-e^{\left(-K\,t\right)}\right]$$

where *C*_∞_ represents the maximum glucose content (mg/mL), and *K* represents the kinetic constant. The area under the hydrolysis curve (AUC) was calculated using the following equation:


$$A\,U\,C=C_{\infty }\times \left(t_{f}-t_{0}\right)-\left(C_{\infty }/K\right)\times \left[1-e^{-K\,\left(t_{f}-t_{0}\right)}\right]$$

where *t*_*f*_ represents the end of the enzymatic hydrolysis reaction (180 min), and *t*_0_ represents the beginning of the enzymatic hydrolysis reaction (0 min). The glycemic index (GI) was calculated from the following equation:


G⁢I=(0.862×c⁢a⁢l⁢c⁢H⁢I×100)+8.198

where calcHI is the starch hydrolysis index, which calculated from AUC_*sample*_/AUC_*white bread*_.

### Statistical analysis

The means ± standard deviation (SD) in triplicate were presented to illustrate the data, ANOVA and Tukey’s test (IBM SPSS Statistics 20) were adopted to analyze the data. Origin 2018 (Origin Lab Corporation, USA) was employed to plot the figures, the significant differences were identified at the *p* < 0.05 and *p* < 0.01 levels.

## Results and discussion

### Total phenolics, total flavonoids, and phenolics profile of kidney beans

Phenolic acids, flavonoids, and anthocyanins are phytochemicals that possess phenolic hydroxyl groups in their molecular structure and provide positive effects on reducing the risk of chronic diseases, including cancer and type II diabetes (29, 30). In the present study, the moisture content of kidney beans was 15.04 ± 0.54% (Y1), 12.94 ± 0.37% (Y2), 13.47 ± 0.45% (Y3), 12.21 ± 0.11% (Y4), 13.80 ± 0.17% (Y5), 12.91 ± 0.11% (Y6), and 14.09 ± 0.69% (Y7), respectively; the total phenolics of the kidney beans ranged from 59.86 ± 2.04 to 579.80 ± 7.96 mg GAE/100 g DW, and the total flavonoids of the kidney beans ranged from 86.16 ± 3.11 to 1008.69 ± 4.50 mg RE/100 g DW, these results were similar to those of mung beans reported by the previous study of which the total phenolics ranged from 1.86 ± 0.01 to 5.07 ± 0.08 mg GAE/g, and total flavonoids ranged from 1.81 ± 0.08 to 5.97 ± 0.23 mg RE/g (31). Y7—black color—presented the highest total phenolics and the second highest total flavonoids. Y3—speckled color—presented the highest total flavonoids and the second highest total phenolics. Y1—white color—presented the lowest total phenolics and total flavonoids. Additionally, Y2, Y4, Y5, and Y6 were claret-colored, greenish brown, red, and atropurpureus-colored, respectively, which presented modest total phenolics and total flavonoids, as shown in Table 1. The regularity of the relations between color and total phenolics and total flavonoids were similar with the previous studies which suggested that the total phenolics and total flavonoids of dark-colored (red, black, and speckled) beans were higher than the white ones (32, 33). Anthocyanins belong to flavonoids, which are the dominant substance appearing various colors, non-white bean coats contained more anthocyanins than the white ones (34). And anthocyanin is constituted by flavonoids with saccharide groups and characterized by the C ring carbon attached to the B ring (35). The substitution pattern of the B ring affects the chromatic features of anthocyanin (36). These phenolics were considered as inhibitor to restrain α-amylase and α-glucosidase (11).

**TABLE 1:** Moisture content and phenolic compounds composition of kidney beans.

Kidney bean varieties	Y1	Y2	Y3	Y4	Y5	Y6	Y7
Color	White	Claret-colored	Red speckled	Greenish brown	Red	Atropurpureus	Black
Moisture content (%)	15.04 ± 0.54 a	12.94 ± 0.37 cd	13.47 ± 0.45 bc	12.21 ± 0.11 d	13.80 ± 0.17 bc	12.91 ± 0.11 cd	14.09 ± 0.69 b
Total phenolics (mg GAE/100 g DW)	59.86 ± 2.04 f	437.06 ± 17.88 c	521.55 ± 13.99 b	390.27 ± 19.51 de	372.47 ± 19.79 e	410.32 ± 8.29 d	579.80 ± 7.96a
Total flavonoids (mg RE/100 g DW)	86.16 ± 3.11 g	673.03 ± 5.34 c	1008.69 ± 4.50 a	383.85 ± 18.88 f	496.42 ± 9.19 e	606.62 ± 13.52 d	758.92 ± 15.89 b
Gallic acid (mg/100 g DW)	39.62 ± 4.45 c	62.48 ± 4.83 b	28.95 ± 4.96 c	37.47 ± 7.00 c	41.90 ± 2.55 c	27.93 ± 3.77 c	118.69 ± 9.21 a
Chlorogenic acid (mg/100 g DW)	3.13 ± 0.16 d	6.25 ± 0.34 c	1.24 ± 0.21 e	5.58 ± 0.30 c	9.52 ± 0.18 b	12.85 ± 1.87 a	7.14 ± 0.69c
2,3,4-trihydroxybenzoic acid (mg/100 g DW)	nd	4.55 ± 0.40 a	2.27 ± 0.40c	2.11 ± 0.10c	3.41 ± 0.16 b	2.18 ± 0.21 c	nd
*p*-hydroxybenzoic acid (mg/100 g DW)	nd	nd	5.50 ± 0.72 c	6.11 ± 0.39 c	10.35 ± 0.34 b	13.91 ± 2.33 a	nd
Catechin (mg/100 g DW)	nd	nd	10.95 ± 0.49 a	10.08 ± 0.32 b	4.34 ± 0.17 c	nd	nd
Ferulic acid (mg/100 g DW)	nd	nd	nd	nd	0.39 ± 0.02 a	nd	nd
Rutin (mg/100 g DW)	nd	nd	nd	nd	28.47 ± 0.90 a	nd	nd

All data were expressed as the means ± SD, *n* = 3. Values in the same row with different letters indicate significant differences (*p* < 0.05). nd, not detected. Y1, Weining white kidney bean; Y2, Biyun no.7; Y3, Qian yundou no.1; Y4, YJ009727A; Y5, Biyun1902; Y6, Biyun no.6; Y7, Biyun no.3.

The individual phenolic compounds of the kidney bean were determined as well. Gallic acid was the most prevalent phenolic compound that ranged from 27.93 ± 3.77 to 118.69 ± 9.21 mg/100 g DW, and Y7 presented the highest content. Chlorogenic acid, 2, 3, 4-trihydroxybenzoic acid, *p*-hydroxybenzoic acid and catechin were minor phenolic compounds found in kidney beans. Ferulic acid (0.39 ± 0.02 mg/100 g DW) and rutin (28.47 ± 0.90 mg/100 g DW) were only present in Y5, as shown in Table 1. Previous studies have shown that gallic acid, ferulic acid, catechin, and rutin were present in beans, with gallic acid being the major compound (8, 37, 38). The content of ferulic acid, catechin and *p*-hydroxybenzoic acid in the present research were higher than those in lentils reported by Zhang (10). Chlorogenic acid is an ester of quinic acid and caffeic acid (39), which may reduce the risk of cardiovascular diseases, obesity and diabetes by lowering reactive oxygen species (35, 40). Moreover, phenolics possess the potential in dietary strategies via the inhibition of α-amylase activity. It has been proposed that phenolics bind to α-amylase through non-covalent interactions such as hydrogen bonds or hydrophobic interactions to form soluble or insoluble polyphenol-protein aggregates, leading to the denaturation or inactivation of α-amylase (41).

### Alpha-amylase inhibitor activity of kidney beans

**FIGURE 2:**
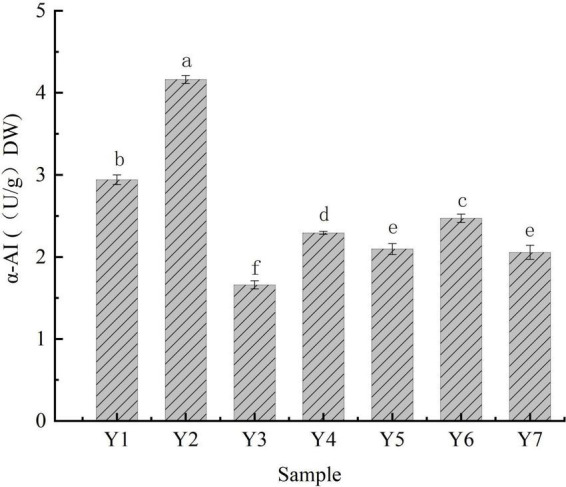
α-AI-specific activities of kidney beans. Bars with different letters indicate significant differences (*p* < 0.05).

The hydrolytic catalysis of α-amylase on the internal α-1,4 glycosidic bond of the molecular chains in starch is critical for starch hydrolysis (42), which results in the hydrolysis of starch into maltose and other oligosaccharides that are further hydrolyzed into glucose by α-glucosidases, eventually, it is absorbed by the gut (43). α-AI is a glycoprotein that inhibits α-amylase activity, resulting in a lower glycemic index (13). The α-AI specific activities of the kidney beans ranged from 1.659 ± 0.050 to 4.162 ± 0.049 U/g DW, Y2 showed the highest activity, followed by Y1, Y6, Y4, Y5, Y7, and Y3, respectively (Figure 2). Beans are an abundant source of α-AI (44, 45). Zhang et al. investigated the α-AI activity in beans (lentil) and found the inhibition concentration of 50% ranged from 23.08 to 42.15 mg/mL in α-amylase (10); moreover, the α-AI-specific activity of rice beans ranged from 7.529 to 10.766 U/g (46). These acquired results of α-AI activity indicate that beans possess potential for use in dietary strategy.

### Starch composition of kidney beans

Starch can be divided into rapidly digestible starch (RDS), slowly digestible starch (SDS) and resistant starch (RS). RDS can be digested within 20 min, SDS can be digested between 20 and 120 min, and RS cannot be digested even after 120 min (25). Compared to RDS, SDS is slowly digested in the digestion system; hence, it keeps stable in the gastrointestinal tract for a longer time and presents minor effects on the stimulation of postprandial insulin-secretion, which maintains the function and sensitivity of insulin, avoiding metabolic syndromes such as hyperinsulinemia and insulin resistance (47, 48). In addition, RS cannot be digested in the small intestine and widely be regarded as a beneficial carbohydrate, especially for glycemic control (47, 49, 50). In the present study, the contribution of starch content to the dry weight of the kidney beans ranged from 28.66 ± 0.37 to 37.98 ± 1.44%, RDS, SDS and RS contributed to the total starch content ranged from 7.80 ± 0.35 to 13.67 ± 0.26%, 5.73 ± 0.41 to 16.31 ± 0.45%, and 70.90 ± 0.39 to 83.12 ± 0.42%, respectively, as shown in Table 2. These results indicated that kidney beans may possess potential for use in glycemic control.

**TABLE 2:** Starch components of kidney beans.

Sample	TS (%)	RDS (%)	SDS (%)	RS (%)
Y1	35.47 ± 0.48 bc	11.16 ± 0.29 e	5.73 ± 0.41 e	83.12 ± 0.42 a
Y2	37.98 ± 1.44 a	12.09 ± 0.54 cd	14.83 ± 0.70 b	73.08 ± 0.46 e
Y3	28.66 ± 0.37 e	13.67 ± 0.26 a	13.99 ± 0.32 b	72.34 ± 0.40 e
Y4	33.96 ± 0.87 cd	7.80 ± 0.35 f	10.32 ± 0.26 c	81.88 ± 0.46 b
Y5	32.43 ± 0.59 d	13.21 ± 0.38 ab	7.96 ± 0.40 d	78.83 ± 0.02 c
Y6	36.62 ± 0.40 ab	12.79 ± 0.15 bc	16.31 ± 0.45 a	70.90 ± 0.39 f
Y7	34.69 ± 0.54 c	11.64 ± 0.28 de	10.82 ± 0.51 c	77.54 ± 0.37 d

All data were expressed as the means ± SD, *n* = 3. Results in the same column with different letters indicate significant differences (p < 0.05). TS, total starch; RDS, rapidly digestible starch; SDS, slowly digestible starch; RS, resistant starch. The values in TS column indicate the contribution of total starch content to the dry weight of kidney beans, the values in RDS, SDS, and RS columns indicate the contribution of the rapidly digestible starch, slowly digestible starch and resistant starch content to the total starch, respectively.

### Glycemic index of kidney beans

Glycemic index (GI) was computed in accordance with the ratio of blood glucose elevating effects of a food to the reference substance, which reflects the blood glucose levels after ingestion of a food (4, 5). The GI of foods can be categorized as three level: low (≤55), medium (55 < GI ≤ 70) and high (>70) (16, 51).

**FIGURE 3:**
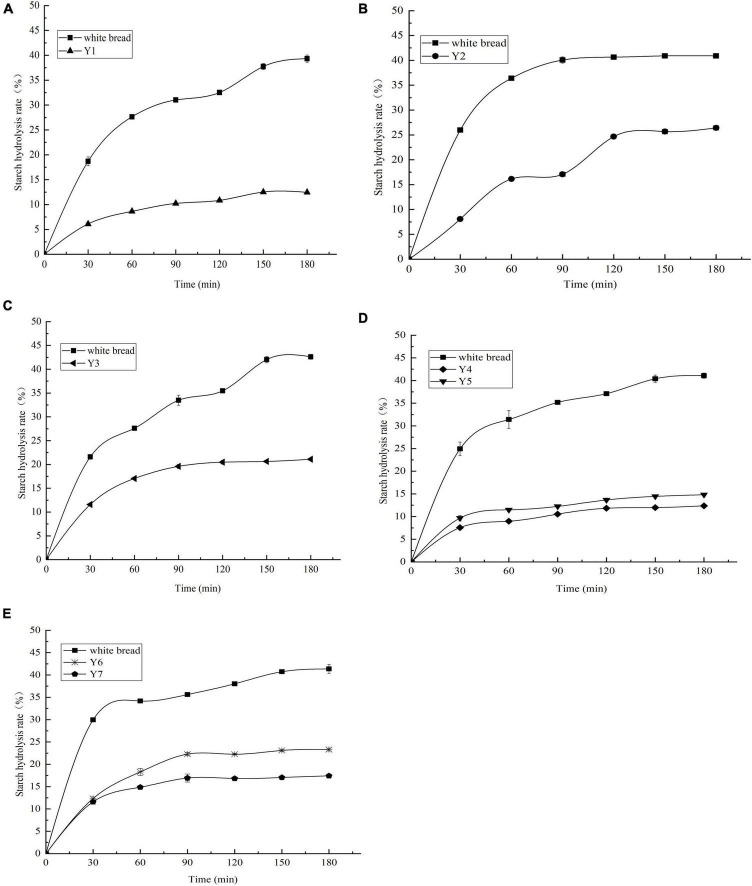
**(A–E)** Starch hydrolysis curves of kidney beans and white bread. Y1, Weining white kidney bean; Y2, Biyun no.7; Y3, Qian yundou no.1; Y4, YJ009727A; Y5, Biyun1902; Y6, Biyun no.6; Y7, Biyun no.3.

In the present study, the GI was implemented in an *in vitro* digestion model coupled with the DNS colorimetry method. Starch hydrolysis curves were plotted according to the SR and hydrolysis time, as shown in Figure 3. The SR of kidney beans was significantly lower than that of white bread (reference). The SR of Y2 was 26.41 ± 0.41% at the end of the digestion period (180 min), followed by Y6, Y3, Y7, and Y5. Y1 and Y4 presented a lower SR, with the values of 12.47 ± 0.45% and 12.37 ± 0.20%, respectively. The kidney beans could be categorized into three groups according to their GIs: Y1, Y4, and Y5 were less than 40 (32.47 ± 0.13–37.66 ± 0.64); Y2 and Y7 were less than 50 (42.87 ± 0.58–49.18 ± 0.42), and Y3 and Y6 were less than 55 (52.48 ± 0.53–52.99 ± 0.56), Y4 had the lowest GI amongst these kidney beans, with the value of 32.47 ± 0.13, as shown in Table 3. The GI of other beans has been evaluated in previous studies, in which the values ranged from 12.00 ± 0.10 to 57.59 ± 3.41 (7, 12, 16, 52–54). Additionally, the RS content manifests extremely negative correlation with the GI (*p* < 0.01, *r* = 0.974), which suggested that the RS makes a dominant contribution to the low GI in kidney beans, this may be due to the RS compositions—insoluble, soluble dietary fiber, and non-digestible oligosaccharides—which cannot be digested in the small intestine (55). These ingredients confer kidney beans with excellent glucose-lowering potential. In the present study, the GI range of kidney beans was 32.47 ± 0.13 ∼ 52.99 ± 0.56 which indicated that they can be categorized into low GI and served as ingredients in functional low GI foods.

**TABLE 3:** Glycemic index (GI) and related indexes of kidney beans.

Sample	SR_(180 *min*)_ (%)^a^	C_∞_^b^	K^c^	AUC^d^	calaHI^e^	GI^f^
Y1	12.47 ± 0.45 f	0.2192 ± 0.0018 f	0.02 ± 0.00 c	28.50 ± 0.09 g	30.48 ± 0.09 e	34.47 ± 0.08 e
Y2	26.41 ± 0.41 a	0.5523 ± 0.0095 a	0.01 ± 0.00 d	54.53 ± 0.57 b	47.55 ± 0.49 b	49.18 ± 0.42 b
Y3	21.10 ± 0.28 c	0.3668 ± 0.0053 c	0.03 ± 0.00 b	52.46 ± 0.65 c	51.96 ± 0.65 a	52.99 ± 0.56 a
Y4	12.37 ± 0.20 f	0.2091 ± 0.0032 f	0.03 ± 0.00 b	29.94 ± 0.16 f	28.16 ± 0.15 f	32.47 ± 0.13 f
Y5	14.81 ± 0.29 e	0.2438 ± 0.0018 e	0.03 ± 0.00 a	36.34 ± 0.79 e	34.18 ± 0.74 d	37.66 ± 0.64 d
Y6	23.31 ± 0.31 b	0.4098 ± 0.0036 b	0.03 ± 0.00 b	57.66 ± 0.69 a	52.18 ± 0.61 a	52.48 ± 0.53 a
Y7	17.41 ± 0.32 d	0.2976 ± 0.0020 d	0.04 ± 0.00 a	45.32 ± 0.76 d	40.23 ± 0.68 c	42.87 ± 0.58 c
W B ^g^	41.06 ± 1.18	0.7431 ± 0.0303	0.03 ± 0.00	105.62 ± 8.08	100.00 ± 0.00	94.40 ± 0.00

All data were expressed as means ± SD, *n* = 3. Results in the same column with different letters indicate significant differences (*p* < 0.05). ^a^SR_(180 *min*)_ (%) indicates the starch hydrolysis rate of the end of the digestion period (180 min). ^b^C_∞_ indicates the maximum glucose content (mg/mL). ^c^K indicates the kinetic constant. ^d^AUC indicates the area under the hydrolysis curve. ^e^calcHI indicates the starch hydrolysis index. ^f^GI indicates the glycemic index. ^g^WB indicates white bread.

## Conclusion

After thorough evaluation of the functional components profile and the potential blood glucose response of kidney beans, it is determined that these kidney beans contain abundant phenolic compounds, in which the phenolics and flavonoids content of dark-colored beans (red, black, and speckled) are higher than the white ones. Gallic acid is the most prevalent phenolic compound, of which the highest content is present in black kidney bean. In addition, these kidney beans which are prone to inhibit α-amylase activity and show resistant properties during the digestion can be categorized as low GI foods. RS makes the dominant contribution to the low GI of kidney beans. The three kidney beans—greenish brown, white, and red—should be emphasized as their GIs present less than 40. These results indicate that kidney beans can be served as ingredients in functional low GI foods for the dietary regulation of type II diabetes patients.

## Data availability statement

The original contributions presented in this study are included in the article/Supplementary material, further inquiries can be directed to the corresponding author.

## Author contributions

YZ: conceptualization, methodology, supervision, and project administration. YZ and SX: validation, data curation, writing—original draft preparation and review and editing, and visualization. SX and MM: formal analysis. YZ, SX, and LQ: investigation. YZ and LQ: resources and funding acquisition. All authors have read and agreed to the published version of the manuscript.

## Abbreviations

GI: glycemic index
α-AI: α-amylase inhibitor
TS: total starch
RDS: rapidly digestible starch
SDS: slowly digestible starch
RS: resistant starch
DNS, 3: 5-dinitrosalicylic acid.
