# Supplementary figures and images for: Functional components profile and glycemic index of kidney beans
Source: Front Nutr. 2022 Nov 2;9:1044427. doi: 10.3389/fnut.2022.1044427 (PMC9667044; doi:10.3389/fnut.2022.1044427)

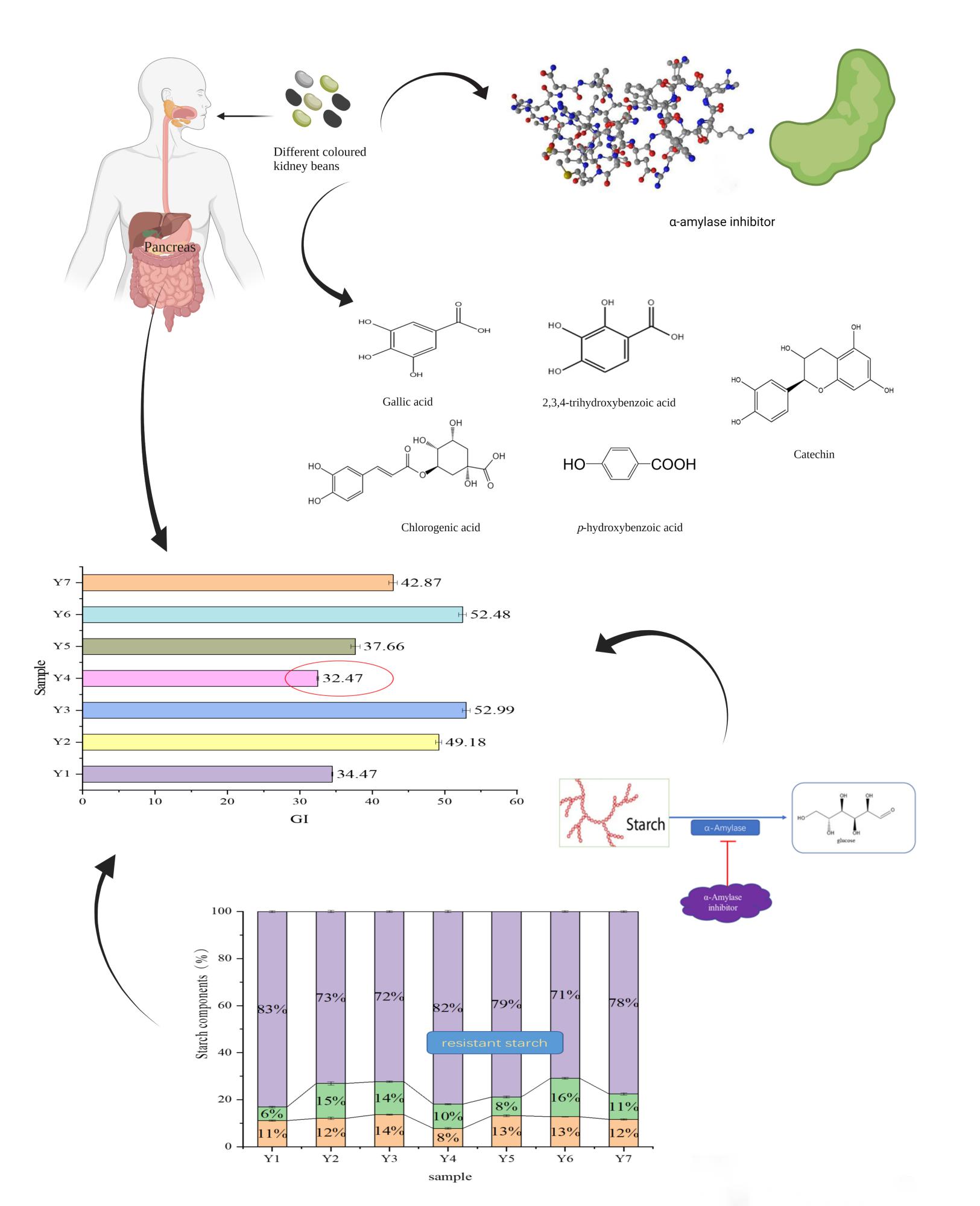

Supplement: Supplementary file 1 [file Image_1.JPEG]
